# Surface-Enhanced Raman Spectroscopy Using a Silver Nanostar Substrate for Neonicotinoid Pesticides Detection

**DOI:** 10.3390/s24020373

**Published:** 2024-01-08

**Authors:** Norhayati Abu Bakar, Marco Fronzi, Joseph George Shapter

**Affiliations:** 1 Australian Institute for Bioengineering and Nanotechnology, University of Queensland, St. Lucia, Brisbane, QLD 4072, Australia; 2 Institute of Microengineering and Nanoelectronic, Universiti Kebangsaan Malaysia, UKM Bangi, Selangor 43600, Malaysia; 3 School of Chemical and Biomedical Engineering, University of Melbourne, Parkville, VIC 3010, Australia; marco.fronzi@gmail.com

**Keywords:** neonicotinoid pesticides, pesticide monitoring, SERS, silver nanostars

## Abstract

Surface-enhanced Raman spectroscopy (SERS) has been introduced to detect pesticides at low concentrations and in complex matrices to help developing countries monitor pesticides to keep their concentrations at safe levels in food and the environment. SERS is a surface-sensitive technique that enhances the Raman signal of molecules absorbed on metal nanostructure surfaces and provides vibrational information for sample identification and quantitation. In this work, we report the use of silver nanostars (AgNs) as SERS-active elements to detect four neonicotinoid pesticides (thiacloprid, imidacloprid, thiamethoxam and nitenpyram). The SERS substrates were prepared with multiple depositions of the nanostars using a self-assembly approach to give a dense coverage of the AgNs on a glass surface, which ultimately increased the availability of the spikes needed for SERS activity. The SERS substrates developed in this work show very high sensitivity and excellent reproducibility. Our research opens an avenue for the development of portable, field-based pesticide sensors, which will be critical for the effective monitoring of these important but potentially dangerous chemicals.

## 1. Introduction

Neonicotinoid pesticides, such as clothianidin, imidacloprid and thiamethoxam, have been restricted in the European Union but remain in use worldwide in the agricultural sector to meet the demands of population growth, urbanisation and market integration. Other neonicotinoid types commonly used to control pests are thiacloprid, dinotefuran, acetamiprid and sulfoxaflor. The demand for neonicotinoid pesticides for crop protection represents almost one-third of the global insecticide market due to their high insecticidal efficiency [1]. To control pests and reduce disease pressure on crops, the use of these pesticides as seed coatings in flowering crops is a widespread practice that can reduce overspray and spray drift. However, the pesticides used to treat the seeds can potentially be absorbed and spread through the tissue and reach pollen and nectars [2]. Then, insect pollinators such as honeybees and wild bees are exposed to the pesticides and bring them to their colonies [3]. The European Union announced a ban on the use of these chemicals after assessment studies from the European Food Safety Authority (EFSA) showed the negative effects of neonicotinoids on insect pollinators, including multiple responses on learning, memory performance and the feeding activity of bee species [4].

Neonicotinoid pesticides have similar chemical structures to nicotine and permanently bind with high infinity to nicotinic acetylcholine receptors in the insect central nervous system [5]. The active substances are neurotoxic and can overstimulate and disrupt the learning and memory behaviour of insect pollinators, resulting in brain damage. These negative impacts on the insect pollinators means they do not interact with crops as expected, affecting the yield and quality of the agricultural products, nutrient content and shelf life. Consequently, the decreasing value of pollinators’ services to agricultural systems leads to big effects on ecosystems and the world economy. Human and environmental exposure to the pesticides through release into the air, diffusion into water and relocation from seed to root, plant and nectar can mean that the pesticides linger for decades in the food chain, animals, humans and the environment. The impact of neonicotinoid pesticides on human health was investigated by Zhang’s group who reported neonicotinoid pesticide residue can be absorbed by the human body through surface water [6]. This can lead to memory loss, finger tremors, headaches, coughs, general fatigue and abdominal pain, as reported by Taira et al., who studied the neonicotinoid exposure in urine samples [7]. Hence, neonicotinoids are compounds of great concern because the active ingredients are systemic, persistent and have high solubility. Therefore, consistent monitoring and rigorous testing of pesticide residue are critical in all countries, especially developing countries.

Effective methods to detect the presence of pesticide residue in food and drinking water has been introduced owing to the increasing public awareness and government concern regarding pesticides’ impact on food security and human health. Tremendous techniques have been established for pesticide monitoring, such as liquid chromatography-mass spectroscopy (LC-MS), gas chromatography–mass spectroscopy (GC-MS), enzyme-linked immunoabsorbent assays and capillary electrophoresis. The Rawat group detected imidacloprid, clothianidin, acetamprid and thiamethoxam in vegetables with a limit of the detection (LOD) at 0.5 µg/mL using high-performance liquid chromatography (HPLC) fitted with a UV-Vis detector [8]. The detection of thiacloprid with an LOD as low as 0.003 ng/mL using a time-resolved fluorescent microsphereimmunochromatographic test strip was reported by the Xu group by using recombinant antibodies [9]. Capillary electrophoresis methods with tandem mass spectroscopy were studied by Carbonell-Rozas’ group for the detection of nine neonicotinoid pesticides with an LOD as low as 1.25 × 10^−5^ g/mL [10]. Generally, these techniques have high sensitivity and are established as a major tool for pesticide identification and qualification. But these analytical techniques are laborious and time consuming, costly, require sophisticated instruments and experienced staff. Many developing countries cannot afford the expensive instruments or do not own sufficient instruments for the monitoring of pesticide residue in food or the environment. In contrast, surface-enhanced Raman spectroscopy (SERS) offers faster analysis time, simpler protocols, low-cost operations and a field-deployable methodology for the identification and qualification of pesticide contamination. Several handheld Raman instruments are available from companies, such as Agilent Technologies, Bruker, Metrohm and Thermo Scientific, for the detection of low concentrations of materials typically in forensic situations. The production of commercial SERS substrates as ultrasensitive detectors as a sensing platform was developed by Nanova Inc. (Columbia, MO, USA), Hamamatsu Photonics (Hamamatsu city, Japan) and Renishaw Diagnostics (Glasgow, UK). However, this work seeks to establish an ultrasensitive detector that will allow the cheap, efficient, reliable mass production of sensing platforms, which would find applications in the detection of a wide range of toxins and other harmful chemicals. The outcomes will be precursors to significant steps towards the production of commercial sensors, which would help developing countries monitor a variety of environmentally harmful chemical species.

The discovery of Raman signal enhancement has opened the door for the first monitoring of single molecules in bioanalytics [11] and pesticides using surface-enhanced Raman spectroscopy (SERS) by simply adsorbing organophosphorus pesticides on a silver surface as a SERS-active substrate [12]. Many publications have shown that the SERS effect is a powerful tool for the identification and qualification of pesticide contamination on the basis of their unique vibrational characteristics [13,14,15]. SERS is a highly sensitive technique that enhances the Raman intensities of molecules more than after adsorption on plasmonic nanostructured surfaces [16,17]. Subsequently, SERS has been continuously used to identify pesticide contaminations in aqueous systems [18] and in seawater [19].

In previous work, we synthesized star-shaped silver nanostructures and fabricated SERS substrates to use SERS enhancement of the Raman signal to detect the neonicotinoid imidacloprid. We studied the best distribution of silver nanostars (AgNs) on glass surfaces by varying the number of layers of AgNs thin films. These SERS substrates are promising for use in the field because detection without complex sample preparation is critical for real-world use in analytical facilities or more importantly in the field. Critically, it is important that such a sensor is sensitive to many different pesticides. The residue of the four pesticides tested in this work has become a global issue as their residues have been found in a variety of food products and environmental samples. Many countries have detected the residue of these pesticides in soil [20], water [6] and food products [21]. In the current work, we studied the four neonicotinoid pesticides on AgNs thin films. The pesticides were deposited on the AgNs surface by a drop-casting technique to observe the sensitivity and reproducibility of SERS substrates towards the neonicotinoid pesticides. This AgNs substrate was found to be a good SERS-active substrate for all the molecules, giving large SERS enhancements and excellent reproducibility with a low relative standard derivation of SERS intensity.

## 2. Materials and Methods

### 2.1. Materials

The four neonicotinoid pesticides used for the SERS measurements were imidacloprid-pestanal, (Analytical standard, Sigma-Aldrich (Sydney, Australia)), thiamethoxam-pestanal, (Analytical standard, Sigma-Aldrich (Sydney, Australia)), thiacloprid-pestanal (Analytical standard, Sigma-Aldrich (Sydney, Australia)) and nitenpyram-pestanal, (Analytical standard, Sigma-Aldrich (Sydney, Australia)). The solvent for preparing the pesticide solutions was methanol (Analytical Reagent Grade, Chem-Supply Pty Ltd (Adelaide, Australia)) and deionized water. The chemicals for the preparation of silver nanostars (AgNs) are silver nitrate (≥99.0%, Sigma Aldrich (Sydney, Australia)), sodium hydroxide (≥98.0%, Chem-Supply Pty Ltd (Adelaide, Australia)), hydroxylamine 50 wt. % in water (Sigma Aldrich, Sydney, Australia) and trisodium citrate dehydrate (≥99.0%, Sigma Aldrich (Sydney, Australia)). Toluene, (Analytical Reagent Grade, Chem-Supply Pty Ltd, (Adelaide, Australia)) and n,n-dimethylformamide (≥99.8%, Sigma Aldrich (Sydney, Australia)) were used for the fabrication of monolayer AgNs films. These chemicals were used as received without any further purification.

### 2.2. Methodology

The synthesis of the colloidal silver nanostars (AgNs) and the fabrication of the silver nanostars film were described in our previous article [22]. Colloidal AgNs was prepared by a chemical reduction technique by mixing 2.0 mL of 6.0 × 10^−2^ M hydroxylamine into 2.0 mL of 5 × 10^−2^ M sodium hydroxide. This mixture was stirred, and the solution colour changed to dark grey after adding 9.0 mL of 1 × 10^−3^ M silver nitrate and 0.1 mL of 1% *w*/*v* trisodium citrate dehydrate into the solution. The colloidal suspension was centrifuged at 6000 rpm for 15 min. The pellet was collected and further used for the fabrication of SERS substrates. AgNs SERS substrates were prepared on glass surfaces using a self-assembly technique. The monolayer AgNs film was prepared by mixing 12.0 mL colloidal AgNs and 6.0 mL toluene to obtain the water–oil interface. The monolayer AgNs films were transferred onto the solid substrate by placing the silicon or glass substrate surface under the formed monolayer solution and the substrate was further dried at 60 °C for 1 h in an oven. This process was repeated multiple times to prepare 10 layers of AgNs films on the substrate surfaces.

Neonicotinoid pesticide solutions were prepared at 1 mg/mL concentration by dissolving 1 mg of the pesticides in 0.5 mL of methanol. The mixture was placed in an ultrasonic bath to dissolve the solid. After that, the pesticide solution was added to 0.5 mL of deionized water. This 1 mg/mL pesticide solution was used as the stock solution to prepare seven concentrations, namely 1 × 10^0^, 1 × 10^−1^, 1 × 10^−2^, 1 × 10^−3^, 1 × 10^−4^, 1 × 10^−5^ and 1 × 10^−6^ mg/mL. These various concentrations were prepared by diluting 1 mg/mL of pesticide solutions with the appropriate amount of deionized water. For SERS measurements, the pesticide was dropped and dried on the AgNs surface and bare-glass surface.

The SERS measurements were performed using a Renishaw model InVia micro Raman spectrometer with 785 nm wavelength diode laser. The Raman spectra of the pesticide powders on glass slides were recorded using a 50× objective at 100% of the 167 mW power laser with 5-second integration time for reference spectra. The SERS spectra of the pesticides on the silver nanostars surface were collected using a 50x objective at 1% of the 167 mW power laser with 5-second integration time. SERS sensor properties such as sensitivity and reproducibility towards the pesticide detection were observed in this work. The reusability and stability of the SERS substrate were reported in a previous article [22].

### 2.3. Computional Model

Partial Least Squares (PLS) regression was used to perform multivariate analysis on spectroscopic data. Python codes using the libraries numpy, scikit-learn and scipy were used for SG filtering and PLS regression. The SG filtering was implemented using the savgol_filter function from the scipy.signal module. PLS regression was carried out using the PLSRegression class from the sklearn.cross decomposition module while cross-validation predictions were made using the cross_val_predict function from sklearn.model selection.

A Savitzky–Golay filter was applied to smooth the spectra and calculate the first derivative of the spectra, which is used in the analysis. The optimal number of PLS components and the relevant wavelengths are determined by optimising the R^2^ and mean square error of cross-validated data. To improve the performance of the model, the values of the concentrations were transformed using the logarithm function, which helps to linearize the relationship between the predictor variables (spectra), and the response variable (concentration) reduces the effect of outliers and spreads the values out more evenly across the range of possible values. This improves the stabilization of the response variable and makes the model more sensitive to small changes at low concentrations. The regression graphs were plotted using Microsoft Excel 2010.

## 3. Results

**Figure 1:**
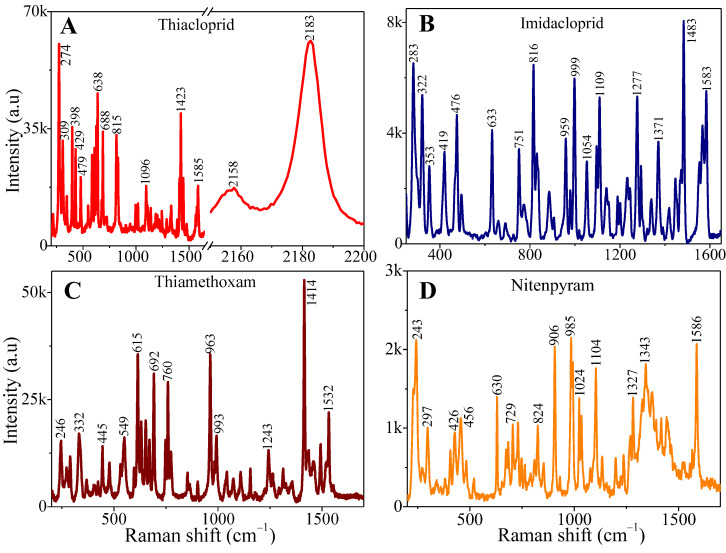
Raman spectrum of the neonicotinoid pesticide powders on bare glass substrates using 100% power laser: (**A**) thiacloprid; (**B**) imidacloprid; (**C**) thiamethoxam; (**D**) nitenpyram.

In this report, we used a AgNs substrate prepared through 10 subsequential layer depositions of silver nanostars (AgNs) on a glass surface as the SERS substrate, as described in our previous article [22]. To comprehensively study the SERS performance for various neonicotinoid pesticides on the SERS substrates, we collected the Raman spectra of neonicotinoid pesticide powders for the reference of neonicotinoid characteristic peaks. Figure 1A–D show the Raman spectra of four neonicotinoid pesticide powders, namely thiacloprid, imidacloprid, thiamethoxam and nitenpyram. The characteristic Raman peaks for each pesticide were obvious. The chemical structures of these pesticides are attached in the SI, Figure S1.

**Figure 2:**
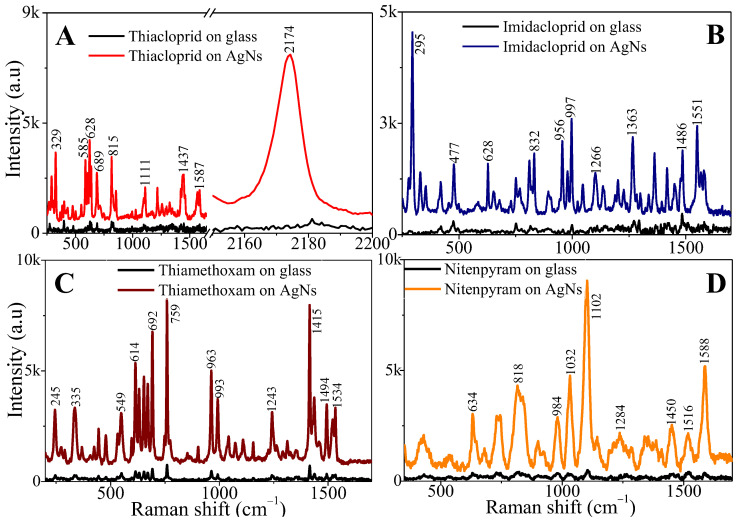
SERS spectra of neonicotinoid pesticides on AgNs surface using 1% power laser. The spectrum on glass surface is provided for reference using 1% power laser. (**A**) thiacloprid; (**B**) imidacloprid; (**C**) thiamethoxam; (**D**) nitenpyram.

The sensitivity of the SERS substrate was studied by observing the Raman spectrum of the pesticides on a glass surface and on the AgNs surface using the same solution concentration for the drop casting and same laser configuration but 100-times less power for the experiments on the SERS substrates. Figure 2A–D display the Raman spectra of the pesticides on the glass surface and on the AgNs surface at a concentration of 1 mg/mL. Based on the SERS spectra, the characteristic peaks of the pesticides on the AgNs surface match those observed for the pesticides on the glass surface. The observed characteristic peaks of the absorbed pesticides on silver nanostars are in good agreement with the observed characteristic peaks of pesticide powders. The details of the characteristic peaks for the pesticide powder, on glass surface and on AgNs, are discussed in the SI, Tables S1–S4. The intensity of the characteristic peaks of these pesticides at 1 mg/mL was clearly enhanced after the absorption of the pesticides on the silver surface compared to the low Raman intensity of the pesticides on bare glass surfaces at 1 mg/mL. To calculate the Enhancement Factor (EF) of SERS performance, some of the highest peaks at 2174 cm^−1^, 295 cm^−1^, 759 cm^−1^ and 1102 cm^−1^ were chosen for thiacloprid, imidacloprid, thiamethoxam and nitenpyram, respectively. The calculated EFs for thiacloprid, imidacloprid, thiamethoxam and nitenpyram detection were 3.61 × 10^5^, 2.86 × 10^4^, 9.53 × 10^4^ and 2.40 × 10^5^, respectively. There are a few EF formulae for EF calculations and, here, we chose the Analytical Enhancement Factor approach, as described by Ru et al.’s group [23]. These EF values were calculated using Formula (1).

(1) $$EF=\frac{I_{SERS}/N_{SERS}}{I_{Raman}/N_{Raman}}$$

where *I_Raman_*: intensity of non-SERS; *I_SERS_*: intensity of SERS; *N_Raman_*: average number of molecule for non-SERS that contributed the signal; and *N_SERS_*: average number of molecule for SERS that contributed the signal.

SERS enhancement happens through two mechanisms, namely an electromagnetic (EM) mechanism and a chemical (CM) mechanism [16,24,25]. The EM mechanism is generally observed on substrates rich in free electrons that can generate localized surface plasmon resonance (LSPR) upon excitation [26]. The CM mechanism can have three possible origins in a metal–molecule system, namely interfacial ground-state charge transfer, photoinduced charge transfer resonance and the electronic excitation resonance within the molecule itself [27]. In our experiments, the laser wavelength is 1.58 eV, while the pesticides examined have energy gaps ranging from 2.084 to 7.94 eV; thus, resonance Raman of the molecule itself is not possible [28]. The Fermi level of silver nanoparticles is generally about −4.26 eV [29], meaning that in all cases, the energy of the LUMO is higher than the Fermi level of AgNs, while the energy level of the HOMO is below the Fermi level. As shown in Table 1, the gaps between the levels for each molecule and the Ag Fermi level vary dramatically but, in all cases, significant SERS enhancement is observed. The large gaps between the LUMOs and the Fermi level mean that a photoinduced charge transfer resonance mechanism is unlikely [29]. Thus, this means that the most likely SERS enhancement mechanism is a combination of electromagnetic enhancement due to surface plasmon excitation in the metal nanoparticles [30] and interfacial ground-state charge transfer. These ground state interactions change the polarizability of the metal–molecule complex, leading to higher Raman cross-sections. Importantly, despite a range of HOMO energy levels of over 2.5 eV, the SERS substrate gives high enhancements for all the pesticides, highlighting the wide applicability of this approach to detect many species of environmental concern.

**Figure 3:**
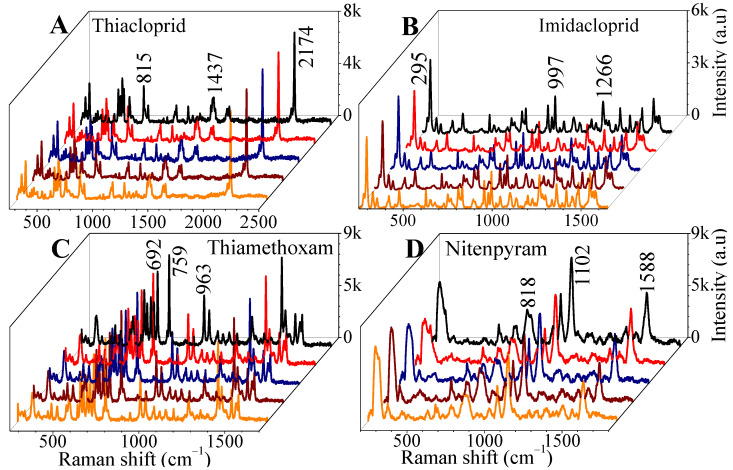
SERS measurement of four pesticides (1 mg/mL) for 5 different spots on the 10 layer AgNs substrate (a complete set of spectra from 20 spots is provided in the SI, Figure S2A). (**A**) thiacloprid; (**B**) imidacloprid; (**C**) thiamethoxam; (**D**) nitenpyram.

The detection reproducibility of the SERS substrate for the four pesticides was tested by collecting the SERS spectra of pesticides at 20 different spots on the same sample surface. Five spectra of five different spots for each of the four pesticides are plotted in Figure 3A–D. However, the full SERS spectra of 20 different spots for four pesticides on AgNs surfaces are provided in the SI, Figure S2. The three highest-intensity characteristic peaks for each pesticide were chosen to calculate the relative standard derivation (RSD) and observe the uniformity field enhancement of the SERS substrate.

**Figure 4:**
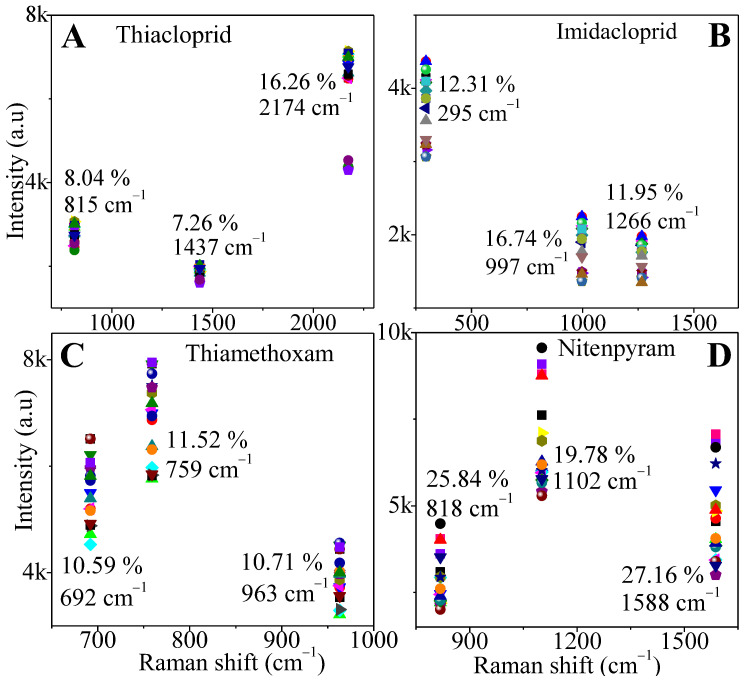
RSD for three peaks of 1 mg/mL four neonicotinoid pesticides on the 10-layer AgNs substrate from the 20 different spots. (**A**) thiacloprid; (**B**) imidacloprid; (**C**) thiamethoxam; (**D**) nitenpyram.

Figure 4A–D show the SERS intensity versus Raman shift for the three highest-intensity characteristic peaks of the pesticides on the AgNs surface at 1 mg/mL concentration. Based on the RSD results, the AgNs created good local field enhancements for the SERS detection of these pesticides by showing major uniform enhancement. The absorption of pesticides on the uniform surface coverage of the silver nanostars clusters with their many spikes yields good SERS reproducibility. The calculated RSD for the four pesticides is as low as 7.26%, and the highest is 27.16%. The SERS substrate showed good reproducibility for thiacloprid detection and the low reproducibility for the nitenpyram detection.

**Figure 5:**
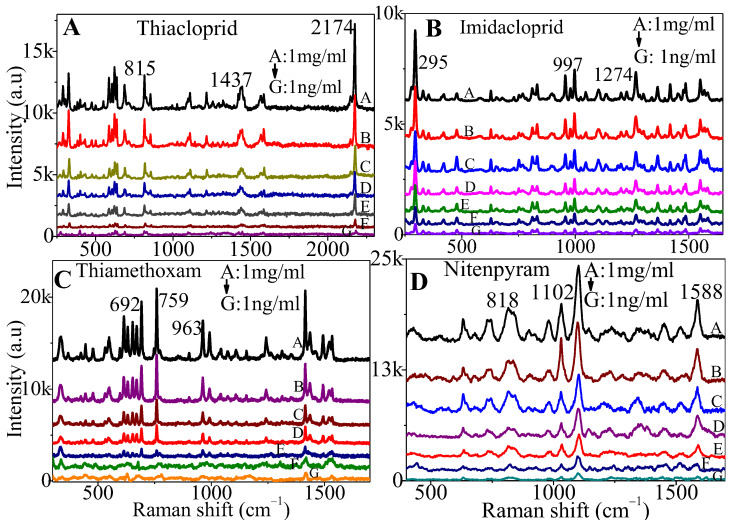
SERS measurement for different concentrations of neonicotinoids on the AgNs surface. (**A**) thiacloprid; (**B**) imidacloprid; (**C**) thiamethoxam; (**D**) nitenpyram.

**Figure 6:**
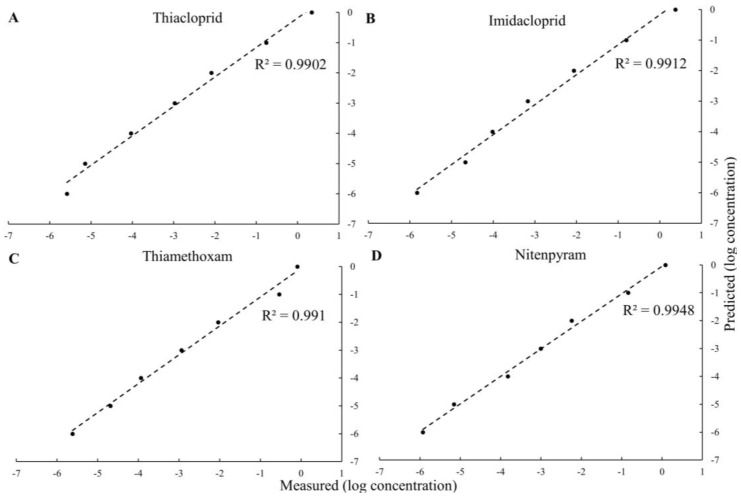
Plots of predicted concentrations vs. actual concentrations in the calibration sets for all four pesticides produced using the PLS regression. (**A**) thiacloprid; (**B**) imidacloprid; (**C**) thiamethoxam; (**D**) nitenpyram.

We further studied the limit of detection of the SERS substrate for these pesticides. Figure 5A–D plot the SERS spectra for different concentrations, ranging from 1 ng/mL to 1 mg/mL of the four pesticides on the AgNs surface. We found that, as expected, a decreasing pesticide concentration gave decreasing SERS intensities. The characteristic peaks of these pesticides at lowest concentration on the AgNs surface are still observable. Using a signal-to-noise ratio of 2 to 1, the limits of detection (LOD) for thiacloprid, imidacloprid, thiamethoxam and nitenpyram were determined and found to be 1.72 × 10^−6^ mg/mL, 9.4 × 10^−8^ mg/mL, 4.83 × 10^−6^ mg/mL and 7.65 × 10^−7^ mg/mL, respectively, on the AgNs surface. The results from the Partial Least Squares (PLS) regression show excellent agreement for all pesticides tested, as shown in Figure 6A–D. These good correlations for these four pesticides show that the SERS substrates could be used to estimate pesticide residue in the environment and food products at very low concentrations, as required by the current limits set by regulatory agencies.

## 4. Discussion

The use of silver nanostars has not previously been observed for the detection of thiacloprid, thiamethoxam and nitenpyram pesticides. However, the detection of imidacloprid has been reported using a silver nanoflower [35] and gold-coated silver nanoflower SERS substrate [14]. The sensitivity observed using AgNs is similar to that observed with silver nanoflowers in terms of the limits of detection for imidacloprid using SERS substrates. However, the addition of gold to silver nanoflowers has been shown to improve the sensitivity by a factor of approximately 100. Other recent reports have used roughened silver [13] and palladium nanoparticles on meso-porous silicon [36] as SERS substrates, and they have exhibited very similar limits of detection for imidacloprid. This finding is beneficial for estimating the imidacloprid residue in food products, since the result is significantly lower than the current allowable limit of residue in fruit by the Chinese government, set at 1.91–3.91 mol/L (4.89 × 10^−4^ mg/mL to 10.0 × 10^−4^ mg/mL), and by the European Union at 0.5 mg/kg (5 × 10^−4^ mg/mL) [15].

A combination of silver and gold nanoparticles has been reported to detect thiacloprid with a limit of detection of 9.6 × 10^−2^ mg/mL [37], 2.3 × 10^−5^ mg/mL in milk [38], 1.0 × 10^−5^ mg/mL in peach [39] and 4.0 × 10^−4^ mg/mL using silver and gold hydrosols [40]. Interestingly, the thiacloprid detected using AgNs is significantly lower than reported by other researchers, and the current allowable limit of residue in honey by the European Union is 200 µg/kg (2 × 10^−4^ mg/mL) [40]. Thiamethoxam has been detected using silver nanoparticle films at a limit of 3.7 × 10^−1^ mg/mL [41], 3.0 × 10^−4^ mg/mL [42] and 3.0 × 10^−6^ mg/mL in fruit by silver nanoparticles decorated with cellulose and DNA [21]. Meanwhile, a gold-embedded chitosan SERS substrate had a similar LOD to this project at 1.0 × 10^−6^ mg/mL on fruit peels [43]. This value is significantly lower than the current allowable limit of thiacloprid residue in fruit by the United States Environmental Protection Agency of 2.0 × 10^−5^ mg/mL to 6.0 × 10^−3^ mg/mL [43]. One article reporting the use of a silver dendrite with a fern structure as the SERS substrate to detect nitenpyram in apple surfaces had an LOD of 1.2 × 10^−3^ mg/mL [44]. The AgNs used in this project provided a more sensitive surface for low concentrations of nitenpyram. We also provide, in Table 2, the wider range of previous works toward the detection of pesticides, chemical traces and biomaterials in complex samples.

## 5. Conclusions

We detected four neonicotinoid pesticides on a silver nanostars (AgNs) surface using surface-enhanced Raman scattering. The developed SERS substrates with a dense coverage of the AgNs on a glass surface in this work show very high sensitivity and excellent reproducibility towards four types of neonicotinoid pesticides (thiacloprid, imidacloprid, thiamethoxam and nitenpyram) in a detection range from 1 ng/mL to 1 mg/mL. The results revealed that the AgNs surface is sensitive to four types of pesticides, and the calculated EFs for thiacloprid, imidacloprid, thiamethoxam and nitenpyram detection were 3.61 × 10^5^, 2.86 × 10^4^, 9.53 × 10^4^ and 2.40 × 10^5^, respectively. The range of electronic properties of the four compounds tested suggests that this system will have wide-ranging use for the detection of many molecules. The use of silver nanostars as a SERS-active element to detect these four neonicotinoid pesticides without complex sample preparation is critical for real-world use in analytical facilities or, more importantly, in the field. The data presented clearly detail the sensor sensitivity and reproducibility—all critical for analytical use. Additionally, the very high sensitivity demonstrated means there is no doubt that the sensor would still provide strong signals using a handheld Raman instrument, meaning that use in the field by non-experts is without doubt feasible. Importantly, based on these experiments, the sensor will have a long shelf life and still provide very high sensitivity to a wide range of molecules, making it ideal for field use, where a range of molecular species will be present.

## Figures and Tables

**Table 1:** Energy levels for the pesticides detected using our SERS substrate. The range reported for imidacloprid is due to different conformers of the molecule [31].

Pesticides	LUMO (eV)	HOMO (eV)	∆E (eV)	EF SERS	Ref.
Imidacloprid	−0.83 to −0.96	−8.55 to −8.77	7.63 to 7.94	2.86 × 10^4^	[31]
Thiacloprid	−1.21	−6.68	5.47	3.61 × 10^5^	[32]
Nitenpyram	−3.051	−5.136	2.084	2.40 × 10^5^	[33]
Thiamethoxam	−1.9398	−7.1478	5.208	9.53 × 10^4^	[34]

**Table 2:** The development of the SERS substrate for the determination of the pesticides and chemicals trace in complex samples.

Plasmonic Nanostructure	Sensor Configuration	Analytical Sensitivity	LOD	Analyte	Ref.
Metal nanoparticles (Au and Ag)	Bifuntional molecule@Ag/Au	10^−4^–10^−8^ M	10^−8^ M	Organochlorine pesticides	[45]
AuNPs	AuNPs on mesoporous silica	1–100 ng/mL	10^−12^ M	Pesticides	[46]
AgNPs	AgNPs on glass fiber	10^−1^–10^−10^ M	10^−10^ M	Doxorubicin drug	[47]
Ag nanoplates and AuNPs	Ag nanoplates/AuNPs on aluminium foil	0.05–1000 ppm	34–63 ppb	Melamine and R6G	[48]
AgNPs	TiO_2_@AgNPs	10^−1^–10^−5^ M	10^−5^ M	Acrylamide and crystal violet	[49]
Au nanostars coated silver	Au nanostars@Ag	0.01–5.0 ppm	0.22 ppm	Thiram	[50]
AgNPs and Au NPs	Ag:Au:poly(amidoamine) dendrimer	10^−4^–10^−7^ M	10^−7^ M	Thiram and ziram	[51]
Au@Ag nanocubes and Au@Ag nanocuboids	Au@Ag core-shell	10^−6^–10^−11^ M	10^−11^ M	Thiram	[52]
Au grating surface	Au@metal organic framework (MOF)	10^−6^–10^−14^ M	10^−12^ M	Organophosphorus pesticides	[53]
Branched AuNPs	Snowflake-like AuNPs	10^−5^–10^−9^ mol/L	10^−8^ mol/L	Organophosphorus pesticides	[54]
AuNPs	AuNPs	0.01–10 mg/L	0.01 mg/L	Toxic insecticides	[55]
Au nanostructures	Au@polyoxometalate nanostructures	10^−6^–10^−7^ M	10^−7^ M	Organophosphorus pesticide	[56]
Au nanorods (AuNRs)	Rough AuNRs	0.0005–5 ppm	0.0005 ppm	Thiram	[57]
AgNPs	Cellulose nanofibers@AgNs	1–100 ppm	5 ppm	Thiabendazole	[58]
AuNPs	Alkyne-labeled AuNPs	40 × 10^−6^–1 × 10^−9^ M	10^−8^ M	Heavy-metal ions	[59]
AgNPs	Flower-shaped AgNPs	10^−4^–10^−10^ M	10^−10^ M	Organophosphorus pesticides	[60]
AuNPs	AuNPs	0.01–10 mg/L	10^−5^ g/L	Chlorpyrifos	[61]
AuNPs	AuNPs/porous zirconia layer	10^−2^–10^−6^ M	10^−6^ M	Organophosphates pesticides	[62]
Au nanowaxberry	polydopamine@Au nanowaxberry	100 nM—1 pM	10^−12^ M	Pesticides, pollutants and explosives	[63]
AuNPs	AuNPs	10 ppb–100 ppm	0.3 ppb	Acephate, carbendazim, thiamethoxam and tricyclazole	[64]

## Data Availability

Data will be made available on request.

## Supplementary Materials

The following supporting information can be downloaded at: https://www.mdpi.com/article/10.3390/s24020373/s1, Figure S1: molecule structures for 4 types of neonicotinoid pesticides; Figure S2: SERS measurement of 1 mg/mL of neonicotinoids pesticide for 20 spots on the 10 layers AgNs substrate (A) thiacloprid, (B) imidacloprid, (C) thiamethoxam and (D) nitenpyram; Table S1: the vibrational assignment for the characteristic peaks of thiacloprid powder, SERS signal of thiacloprid and DFT calculation; Table S2: the vibrational assignment for the characteristic peaks of imidacloprid powder, SERS signal of imidacloprid and DFT calculation; Table S3: the vibrational assignment for the characteristic peaks of thiamethoxam powder, SERS signal of thiamethoxam and DFT calculation; Table S4: the vibrational assignment for the characteristic peaks of nitenpyram powder, SERS signal of nitenpyram and DFT calculation.
