# Supplementary material for: Surface-Enhanced Raman Spectroscopy Using a Silver Nanostar Substrate for Neonicotinoid Pesticides Detection
Source: Sensors (Basel). 2024 Jan 8;24(2):373. doi: 10.3390/s24020373 (PMC10820608; doi:10.3390/s24020373)
Supplement: Supplementary file 1 [file sensors-24-00373-s001.zip › sensors-2784261-Supplementary.pdf]

Surface-Enhanced Raman Spectroscopy Using a Silver Nanostar Substrate for Neonicotinoid Pesticides Detection

Norhayati Abu Bakar [1,2,*], Marco Fronzi [3] and Joseph George Shapter [1,*]

[1] Australian Institute for Bioengineering and Nanotechnology, University of Queensland, St. Lucia, Brisbane, QLD 4072, Australia

[2] Institute of Microengineering and Nanoelectronic, Universiti Kebangsaan Malaysia, UKM Bangi, Selangor 43600, Malaysia

[3] School of Chemical and Biomedical Engineering, University of Melbourne, Parkville, VIC 3010, Australia; marco.fronzi@gmail.com

* Correspondence: norhayati.ab@ukm.edu.my (N.A.B.); n.abubakar@uq.edu.au or j.shapter@uq.edu.au (J.G.S.)

Supporting Information:

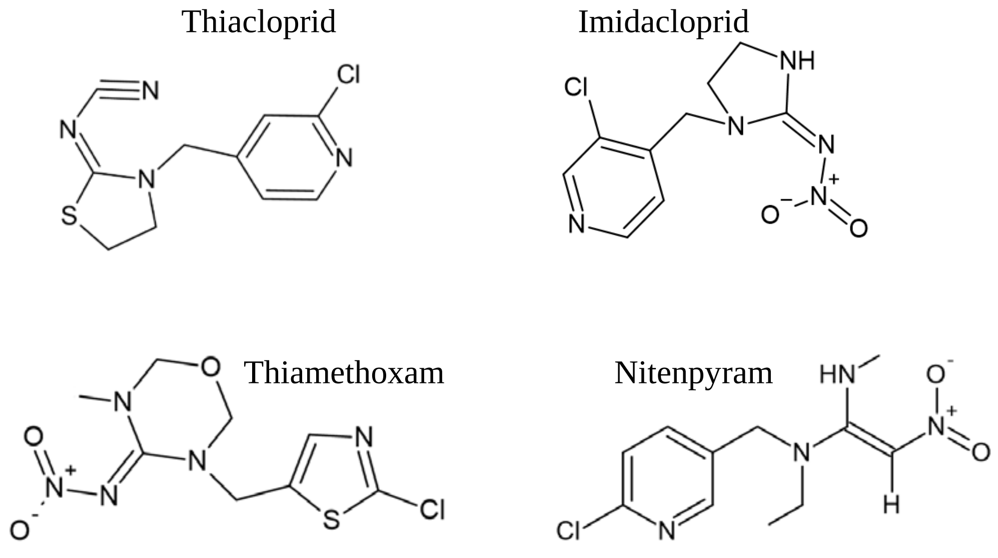

Figure S1: Molecule structures for 4 types of neonicotinoid pesticides

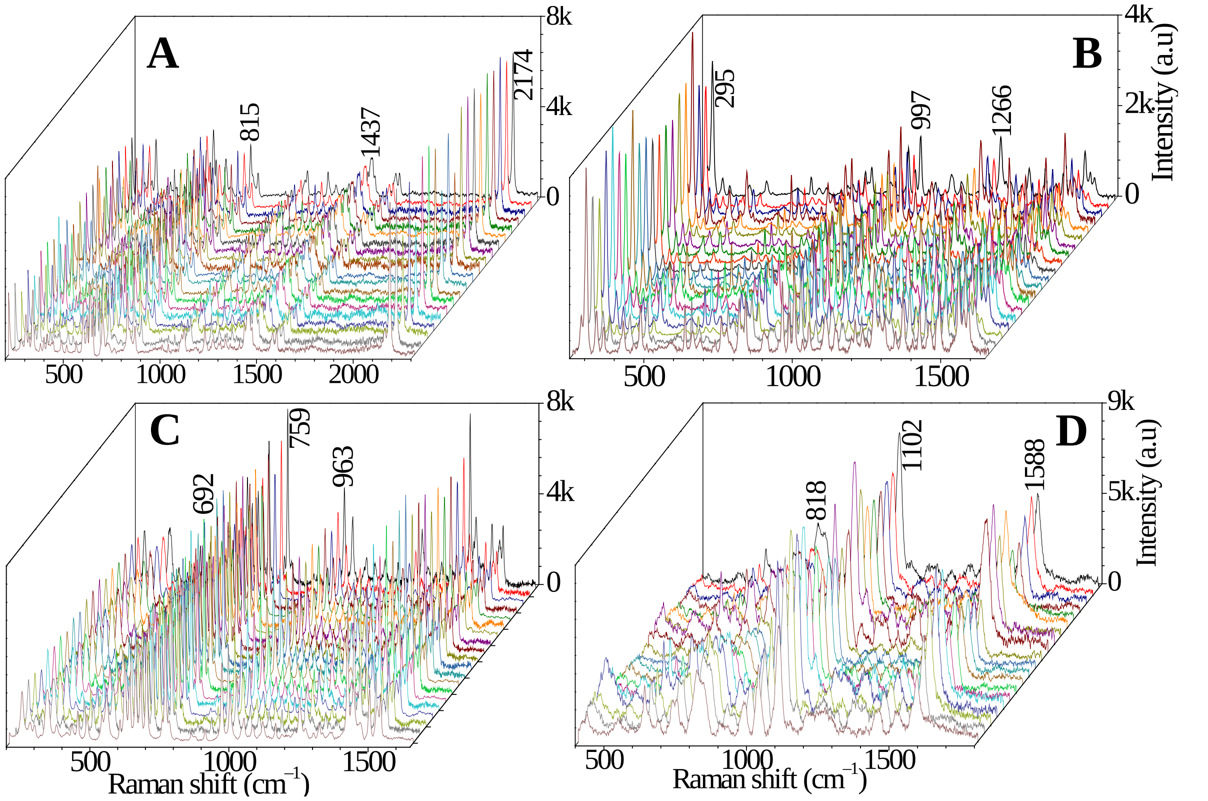

Figure S2: SERS measurement of 1 mg/mL of neonicotinoids pesticide for 20 spots on the 10 layers AgNs substrate (A) thiacloprid, (B) imidacloprid, (C) thiamethoxam and (D) nitenpyram.

Table S1: The vibrational assignment for the characteristic peaks of thiacloprid powder, SERS signal of thiacloprid and DFT calculation.

Experimentally observed peaks		Density Funtional Theory (DFT) (cm^{-1}) [1]	Assignments
Thiacloprid powder (cm^{-1})	SERS (cm^{-1})		
274	289	265	Skeletal vibration
309	329	307	Skeletal vibration
398	399	399	Skeletal vibration
429	432	433	Skeletal vibration
479	475	508	Skeletal vibration
548	550	536	C-N bending
586	585	586	C-N bending, C-S stretching
608	606	610	ring bending, CH_2 rocking
626	628	616	C-Cl bending, C-N bending, C-H bending
638	635	646	C-C bending, C-S stretching
688	689	682	C-S stretching, C-N bending, ring bending, C-Cl stretching
710	708	708	C-S stretching, CH_2 rocking, C-N bending, ring bending
815	815	829	C-H bending, CH_2 rocking, C-N stretching, ring bending, C-Cl stretching
996	995	995	C-N stretching, CH_2 rocking, C-H bending, ring stretching
1019	1025	1046	CH_2 rocking
1096	1111	1082	C-N stretching, CH_2 bending, C-S stretching, CH_2 wagging deformation
1140	1141	1125	C-Cl stretching, ring stretching, C-H bending
1172	1167	1165	C-H bending, ring stretching
1186	1214	1181	C-N stretching, CH_2 bending, ring stretching
1243	1244	1238	CH_2 bending, ring stretching, CH_2 rocking
1289	1287	1278	CH_2 bending, ring stretching, CH_2 rocking
1332	1328	1357	CH_2 bending, ring stretching, CH_2 rocking
1388	1393	1402	CH_2 bending, ring stretching, CH_2 rocking
1423	1422	1429	CH_2 bending
1447	1437	1443	CH_2 bending
1478	1461	1469	CH_2 bending
1563	1569	1577	ring stretching
1585	1587	1595	C=N stretching, N-C-S bending
2183	2174	2185	C≡N stretching

Table S2: The vibrational assignment for the characteristic peaks of imidacloprid powder, SERS signal of imidacloprid and DFT calculation.

Experimentally observed peaks		Density Funtional Theory (DFT) (cm^{-1}) [2]	Assignments
Imidacloprid powder (cm^{-1})	SERS (cm^{-1})		
283	295	274	C-H rocking, C-Cl bending
322	329	306	C-N wagging, C-N bending
476	477	453	C-N rocking, C-N bending, C-Cl stretching, C-C wagging, C-N twisting
633	628	622	C-N bending, C-C bending
751	751	768	N-O bending, C-N bending, C-C stretching, C-H rocking
816	811	815	C-N bending, C-C stretching, C-Cl stretching, C-H rocking
999	997	1008	C-N bending, C-N bending
1109	1106	1126	C-H bending, C-Cl stretching
1142	1140	1197	C-C bending, C-H wagging
1190	1190	1217	C-H twisting
1245	1242	1254	C-C twisting, C-H wagging, C-N stretching, N-N stretching
1277	1266	1280	N-O stretching, C-H twisting, C-H wagging, C-H bending
1295	1300	1282	C-H wagging, C-H bending, N-O stretching
1371	1363	1376	C-C stretching, C-N stretching, C-H wagging
1448	1450	1449	C-C stretching, C-N stretching, C-H bending
1483	1486	1483	C-H bending
1551	1552	1547	C-C stretching
1583	1583	1606	C-C stretching

Table S3: The vibrational assignment for the characteristic peaks of thiamethoxam powder, SERS signal of thiamethoxam and DFT calculation.

Experimentally observed peaks		Density Funtional Theory (DFT) (cm^{-1}) [3]	Assignments
Thiamethoxam powder (cm^{-1})	SERS (cm^{-1})		
423	423	426	H-C-H rocking, C-N-C bending, C-N-C bending
445	447	445	C-Cl stretching, C-S-C bending
478	478	489	C-S-C wagging
530	534	529	C-N-C bending, C-S-C wagging, C-H-C twisting
549	549	545	C-N-C-H wagging, C-H wagging
596	597	601	C-N wagging, H-N-C wagging, C-N-C-S wagging
615	614	616	C-S-Cl stretching
631	631	636	C-S stretching, C-N-C-H wagging, C-N wagging
652	653	653	C-S stretching, C-N-C-H wagging
669	669	686	O-C-N-C wagging, C-N-C-H wagging, C-N wagging
692	692	716	C-H wagging, C-N wagging
759	759	757	C-N-N-O wagging, N-C-N-N wagging
902	902	912	C-H wagging, C-H-C-S wagging, C-H-C-N wagging
963	963	952	C-H scissoring
993	993	983	O-N-O stretching, N-N stretching, H-C-O-C wagging
1043	1042	1053	H-C-N stretching, H-C-N-C wagging, H-C-O-C wagging
1074	1072	1088	C-N stretching, H-C-N scissoring, H-C-N-C wagging
1109	1108	1116	H-C-N scissoring, H-C-N-C wagging
1155	1156	1145	H-C-N scissoring, H-C-N-C wagging
1243	1243	1239	H-C-O scissoring, H-C-N scissoring, H-C-N-C wagging
1314	1312	1315	O-C-H scissoring, H-C-N-C wagging
1358	1351	1369	N-N-C scissoring, O-C-H scissoring, H-C-N-C wagging
1414	1415	1388	H-C-N scissoring, H-C-C scissoring
1436	1435	1435	H-C-O scissoring, H-C-N scissoring

1493	1494	1489	H-C-H scissoring, H-C-N-C wagging
1532	1534	1525	H-C-H scissoring, H-C-O scissoring, H-C-N-C wagging

Table S4: The vibrational assignment for the characteristic peaks of nitenpyram powder, SERS signal of nitenpyram and DFT calculation.

Experimentally observed peaks		Density Funtional Theory (DFT) (cm^{-1}) [4]	Assignments
Nitenpyram powder (cm^{-1})	SERS (cm^{-1})		
630	634	635	C-C-C bending
824	818	856	O-N-O wagging
985	984	996	Ring breathing
1104	1102	1093	C-Cl stretching
1133	1144	1116	C-N stretching
1282	1284	1288	C-C stretching
1442	1450	1465	$-CH_3$ bending, $-CH_2$ scissoring, ring stretching
1518	1516	1515	$-NO_2$ stretching
1586	1588	1602	C=C stretching

References

1. Cañamares, M.V.; Feis, A. Surface-Enhanced Raman Spectra of the Neonicotinoid Pesticide Thiacloprid. *J. Raman Spectrosc.* **2013**, *44*, 1126–1135, doi:https://doi.org/10.1002/jrs.4339.

2. Creedon, N.; Lovera, P.; Moreno, J.G.; Nolan, M.; O'Riordan, A. Highly Sensitive SERS Detection of Neonicotinoid Pesticides. Complete Raman Spectral Assignment of Clothianidin and Imidacloprid. *J. Phys. Chem. A* **2020**, *124*, 7238–7247, doi:10.1021/acs.jpca.0c02832.

3. Gao, Y.; Xu, M.-L.; Xiong, J. Raman and SERS Spectra of Thiamethoxam and the Ag3–Thiamethoxam Complex: An Experimental and Theoretical Investigation. *J. Environ. Sci. Heal. Part B* **2019**, *54*, 665–675, doi:10.1080/03601234.2019.1631099.

4. Wang, Q.; Liu, Y.; Bai, Y.; Yao, S.; Wei, Z.; Zhang, M.; Wang, L.; Wang, L. Superhydrophobic SERS Substrates Based on Silver Dendrite-Decorated Filter Paper for Trace Detection of Nitenpyram. *Anal. Chim. Acta* **2019**, *1049*, 170–178, doi:https://doi.org/10.1016/j.aca.2018.10.039.
